# 

**Published:** 1893-08-19

**Authors:** 


CONTENTS.

A New Order of General Practitioners - - - - - - 321

Around the Hospitals - - - - - - - 322

Diet in Disease. By Mrs. Ernest Hart. XXVIII.—Chronic Bright's Disease - - - - - - - 323

Gleanings in Science and Medicine - - - - - 324

The Apotheosis of the Pawnbroker - - - - - 325

Notes and News - - - - - - - - 328

The Hospital Clinic—

The London Hospital.—Treatment of Compound Fractures - 329

Royal Infirmary, Edinburgh.—The Diagnosis and Treatment of Cystic Tumours in the Scalp - - - - - 330

Notes on the Treatment of Diabetes Mellitus - - - 331

New Drugs and Preparations.—Paraldehyde - - - - 332

The History and Capabilities of Herbal Simples. By W. T. Fernie, M.D.—Garlic and the Onion - - - - - 326

Annotations.—Practical Education—Laundry Work for Feeble-minded Girls—Overcrowding in Lanarkshire - - - - 327

The Editor's Letter-Box - - - - - - - - 332

The Institutional Workshop—

Hospital Administration.—I.—General Hospitals - - - 333

The Story of the Insane from Year to Year - - - 334

Practical Departments.—Ambulance Equipments - - - 335

Construction Notes - - - - - - - - 336

Scraps and Gleanings - - - - - - - - 336

The Book World of Medicine and Science - - - - vii

Medical Vacancies and Appointments - - - - - ix

EXTRA SUPPLEMENT.—THE NURSING MIRROR.

News from the Nursing World.—St. Luke's House—A New Opening—"The Hospital" Competition for Nurses' Homes—Without a Name—An Ample Recognition—Nursing at Winchester—Stout in Proportion—The Control of Nursing—Illinois Training School, Chicago—Other American News—To Be Left Till Called For—Thoroughness—Short Items - cci

Training Schools in America. By Miss Irene Sutliffe cciii

Midwives' Registration - - - - - - - - cciv

Nurses' Homes. I.—Past - - - - - - - - cc

Everybody's Opinion.—Australia or New Zealand?—English Nurses in Paris—Rating Her Sisters—Outdoor Uniforms—The Cape Mission—Dublin—Girl Nurses - - - ccvi

The World of Nurses. As Seen by an Unprofessional Correspondent. I.—Deterioration - - - - - - - ccvi

Death in Our Ranks—Notes and Queries - - - - ccvii

The Muses' Looking Glass.—Medieval Lore - - - ccviii

Nurses in Workhouses - - - - - - - - ccviii

The Book World for Women and Nurses - - - - ccix

